# 合并轻链型淀粉样变性的初诊多发性骨髓瘤患者临床回顾性分析

**DOI:** 10.3760/cma.j.issn.0253-2727.2022.04.011

**Published:** 2022-04

**Authors:** 咏 刘, 红英 尤, 灵芝 颜, 松 金, 京晶 商, 晓兰 施, 霜 颜, 卫芹 姚, 德沛 吴, 蔚 刘, 琤琤 傅

**Affiliations:** 1 苏州大学附属第一医院，江苏省血液研究所，国家血液系统疾病临床医学研究中心，国家卫生健康委员会血栓及止血重点研究室，苏州 215000 Jiangsu Institute of Hematology, National Clinical Research Center for Hematologic Diseases, NHC Key Laboratory of Thrombosis and Hemostasis, The First Affiliated Hospital of Soochow University, Suzhou 215000, China; 2 苏州大学附属第一医院病理科，苏州 215000 Department of Pathology, The First Affiliated Hospital of Soochow University, Suzhou 215000, China

**Keywords:** 多发性骨髓瘤, 淀粉样变性, 临床特征, 预后, Multiple myeloma, Amyloidosis, Clinical features, Prognosis

## Abstract

**目的:**

分析合并轻链型淀粉样变性（AL）的初诊多发性骨髓瘤（MM）患者的临床特征、疗效及预后。

**方法:**

回顾性分析苏州大学附属第一医院自2017年1月1日至2018年10月31日收治的160例初诊MM患者的临床资料。根据患者骨髓、皮肤和其他部位组织病理活检结果是否合并淀粉样变性分为两组，即合并淀粉样变性的MM（MM+AL）组和MM组，比较两组患者的临床特征及疗效。

**结果:**

160例初诊MM患者中，MM+AL组42例，MM组118例。在临床特征方面，MM+AL组的受累轻链与非受累轻链差值（dFLC）明显高于MM组（*P*＝0.039）。诱导治疗后MM+AL组较MM组有更高的总缓解率（85.7％对79.7％，*P*<0.05）和非常好的部分缓解率（76.2％对55.1％，*P*<0.05）。中位随访26（0.25～41）个月，MM+AL组与MM组患者总生存（OS）和无进展生存的差异无统计学意义（*P*值均>0.05），自体造血干细胞移植组患者的OS优于未移植组患者（*P*值均<0.05）。MM+AL组心脏受累患者的预后较MM组和MM+AL组非心脏受累患者差（*P*<0.001），中位OS时间仅为13个月。

**结论:**

MM+AL患者和MM患者的鉴别诊断需行组织病理活检，尤其是dFLC明显增高患者。MM+AL组患者4个疗程诱导化疗后总体缓解率较MM组更高。MM+AL组累及心脏患者预后较差。

## Abstract (en)

**Objective:**

To analyze the clinical characteristics, treatment response, and prognosis of newly diagnosed symptomatic multiple myeloma（MM）patients with systemic light chain amyloidosis（AL）.

**Methods:**

The clinical data of 160 patients with newly diagnosed MM treated at the First Affiliated Hospital of Soochow University from January 1, 2017 to October 31, 2018, were retrospectively analyzed. According to the histopathological biopsy results of bone marrow, skin, and other tissues, the patients were divided into two groups according to whether amyloidosis was combined or not, namely, the MM+AL group and the MM group. The clinical characteristics and treatment responses of the two groups were compared.

**Results:**

Among the 160 patients with newly diagnosed MM, there were 42 cases in the MM+AL group and 118 cases in the MM group. In terms of clinical features, the involved light chain and non-involved light chain（dFLC）in the MM+AL group was significantly higher than that in the MM group（*P*＝0.039）. After induction treatment, the MM+AL group had a higher overall response rate（85.7％ *vs* 79.7％, *P*<0.05）and higher excellent partial response（76.2％ *vs* 55.1％, *P*<0.05）. After a median follow-up of 26（0.25–41）months, there was no significant difference in the progression free survival and overall survival（OS）between the two groups（*P*>0.05）. The OS of patients in autologous hematopoietic stem cell transplantation group was better than that in non transplantation group（*P*<0.05）.The prognosis of patients with cardiac involvement in the MM+AL group was significantly worse than that in the MM group and MM+AL group without cardiac involvement（*P*<0.001）, with a median OS of only 13 months.

**Conclusion:**

The differential diagnosis between the MM+AL and MM groups requires histopathology, particularly for patients with significantly increased dFLC. The overall remission rate of patients in MM+AL group after 4 courses of induction chemotherapy was higher than that in MM group. The prognosis of patients with cardiac involvement in MM+AL group was poor.

多发性骨髓瘤（multiple myeloma，MM）是克隆性的恶性浆细胞肿瘤，主要临床表现由单克隆蛋白、恶性细胞或由恶性细胞分泌的细胞因子驱动，引起典型CRAB症状（包括贫血、肾功能不全、溶骨性损害及高钙血症）和一些不典型并发症，如轻链型淀粉样变性（systemic light chain amyloidosis，AL）[1]。AL是由于轻链型淀粉样蛋白在全身细胞外组织间隙中沉积，从而破坏细胞和器官功能的疾病[2]–[3]。MM和AL虽同为恶性浆细胞疾病，但迄今为止，骨髓瘤相关的AL的发病机制尚不清楚，轻链型淀粉样蛋白的沉积是否会影响MM患者的生物学特征及临床意义，目前也缺少大型多中心前瞻性临床试验对其展开描述。国内外相关研究仅以个案报道为主，临床医师对其总体认识仍然不足，亟需对MM合并AL患者的临床特点及疗效进行分析，提高临床医师的诊治能力。

## 病例与方法

1. 研究对象：本研究收集自2017年1月1日至2018年10月31日，苏州大学附属第一医院确诊的172例初治有症状MM患者的临床资料。MM诊断和分期标准参照2017年修订版中国多发性骨髓瘤诊治指南，其中42例确诊合并AL，均有任一CRAB临床表现，诊断参照病理诊断，至少1个组织病理活检和（或）骨髓活检刚果红染色阳性，且沉积物经免疫组化证实为免疫球蛋白轻链沉积[4]–[6]。

2. 细胞遗传学：所有患者在初次诊断治疗前均采用经CD138分选后的荧光原位杂交（FISH）技术检测细胞遗传学，检测的主要异常指标包括1q21扩增、13q14缺失、Rb1缺失、IgH重排、17p缺失。其中IgH重排阳性者会加做t（4;14）、t（11;14）、t（14;16）检测。根据2018年Mayo Clinic（梅奥医学中心）基于细胞分子遗传学推出的MM预后分层体系mSMART 3.0分期[7]，将细胞遗传学高危定义如下：t（4;14）、t（14;16）、1q21扩增、17p缺失，其他非遗传学高危包括del（13q）/13q14、Rb1缺失、t（11;14）。

3. 疗效评估：诱导治疗后疗效评估参照2017年修订版中国多发性骨髓瘤诊治指南[4]，分为完全缓解（CR）、非常好的部分缓解（VGPR）、部分缓解（PR）、疾病稳定（SD）及疾病进展（PD）。总缓解率（ORR）定义为诱导治疗后疗效达PR及PR以上。AL器官疗效标准参照原发性轻链型淀粉样变的诊断和治疗中国专家共识（2016年版）[6]，分为缓解与进展。

4. 随访：172例患者中9例在治疗及随访期间均无法确定是否合并淀粉样变性，3例在我院行1次治疗后失访，同样无法确定是否合并淀粉样变性，故最终明确诊断的160例患者纳入相关分析。随访截止日期为2020年5月31日。通过查阅住院病历、门诊病历及电话进行随访。对于随访期间死亡的病例，通过病历记录和（或）电话联系患者家属确认。总生存（OS）时间定义为自确诊之日至末次随访或死亡时间。无进展生存（PFS）时间定义为自确诊之日至疾病发生进展或死亡时间。

5. 统计学处理：采用SPSS 25.0软件进行统计学分析。计数资料用例数（百分比）表示，计量资料用*M*（范围）表示。均数比较采用独立样本*t*检验和方差分析的双侧检验，率的比较采用*χ*^2^检验。采用Kaplan-Meier法绘制生存曲线，单因素分析采用Log-rank检验，*P*<0.05为差异有统计学意义。

## 结果

1. 一般资料：纳入分析的160例患者的基本临床特征见表1，MM+AL组受累轻链与非受累轻链差值（dFLC）≥100 mg/L患者所占比例高于MM组患者（85.7％对68.6％，*P*＝0.039），余临床特征的差异均无统计学意义。

**表1:** 多发性骨髓瘤（MM）与MM合并轻链型淀粉样变性（MM+AL）患者的基本临床特征比较

特征	MM组（118例）	MM+AL组（42例）	*t*值/*χ*^2^值	*P*值
年龄［岁，*M*（范围）］	58（38～81）	58.5（42～74）	-0.815	0.416
分型［例（％）］			7.708	0.103
IgA型	26（22.0）	5（11.9）		
IgG型	56（47.5）	19（45.2）		
IgD型	10（8.5）	2（4.8）		
轻链型	23（19.5）	16（38.1）		
其他	3（2.5）	0（0）		
DS分期［例（％）］			1.155	0.282
Ⅰ期、Ⅱ期	6（5.1）	4（9.5）		
Ⅲ期	110（93.2）	36（85.7）		
ISS分期［例（％）］			5.121	0.077
Ⅰ期	25（21.2）	6（14.3）		
Ⅱ期	47（39.8）	11（26.2）		
Ⅲ期	40（33.9）	22（52.4）		
R-ISS分期［例（％）］			5.809	0.055
Ⅰ期	16（13.6）	3（7.1）		
Ⅱ期	65（55.1）	20（47.6）		
Ⅲ期	17（14.4）	13（31.0）		
HGB<100 g/L［例（％）］	71（64.0）	30（73.2）	1.138	0.286
白蛋白≤35 g/L［例（％）］	55（50.5）	13（34.2）	2.992	0.084
血肌酐>177 µmol/L［例（％）］	25（23.1）	15（37.5）	3.048	0.081
血清钙>2.75 mmol/L［例（％）］	5（4.7）	5（12.5）	2.757	0.097
β_2_-微球蛋白>3.5 mg/L［例（％）］	63（61.2）	26（68.4）	0.628	0.428
24 h尿蛋白>5 g［例（％）］	7（7.9）	7（18.9）	3.233	0.072
NT-proBNP>332 ng/L［例（％）］	38（43.7）	17（56.7）	1.511	0.219
dFLC［例（％）］			4.270	0.039
≥100 mg/L	81（68.6）	36（85.7）		
<100 mg/L	28（23.7）	4（9.5）		
细胞遗传学［例（％）］			0.014	0.907
高危	51（43.2）	19（45.2）		
标危	41（34.7）	16（38.1）		
诱导治疗方案［例（％）］			14.850	0.095
以硼替佐米为主的方案^a^	105（88.9）	36（85.7）		
以来那度胺为主的方案^b^	11（9.3）	6（14.3）		
其他^c^	2（2.8）	0（0）		
是否行自体造血干细胞移植［例（％）］			0.248	0.618
是	65（55.1）	25（59.5）		
否	53（45.0）	17（40.5）		

注：NT-proBNP：氨基末端B型钠尿肽原；dFLC：血清受累轻链与非受累轻链差值；a：PAD（硼替佐米+阿霉素+地塞米松）、PDD（硼替佐米+脂质体阿霉素+地塞米松）、PTD（硼替佐米+沙利度胺+地塞米松）、PCD（硼替佐米+环磷酰胺+地塞米松）、PD（硼替佐米+地塞米松）；b：RD（来那度胺+地塞米松）；c：TDD（沙利度胺+脂质体阿霉素+地塞米松）

2. 组织病理资料：在160例患者中有152例（95％）患者进行了骨髓刚果红染色，阳性11例（7.2％）；共有57例行皮下脂肪活检（57/160，35.5％），阳性29例（29/57，50.9％）；仅有1例行肺组织活检且刚果红染色阳性；42例MM+AL患者均完善超声心动图，其中6例存在淀粉样变性心肌累及表现（6/42，14.3％），包含1例心肌组织活检刚果红染色阳性。所有刚果红染色阳性患者均具有AL的病理学依据，即在光镜下呈无定形、均匀的嗜伊红物质，在偏振光显微镜下呈典型的苹果绿色双折光，且沉积物经免疫组化证实为免疫球蛋白轻链沉积。

3. 细胞遗传学异常情况分析：MM组、MM+AL组分别有92例（78.0％）和35例（83.3％）患者在研究中进行经CD138分选后的FISH检测且数据完整。MM组患者1q21扩增、t（4;14）、del（17p）、t（11;14）、del（13q）/13q14、RB1缺失的例数分别为45例（48.9％）、14例（15.2％）、9例（9.8％）、7例（7.6％）、44例（47.8％）、45例（48.9％）。MM+AL组患者1q21扩增、t（4;14）、del（17p）、t（11;14）、del（13q）/13q14、RB1缺失的例数分别为18例（51.4％）、0例（0）、4例（11.4％）、2例（5.7％）、15例（42.9％）、16例（45.7％）。两组患者仅t（4;14）阳性例数的差异有统计学意义（*P*＝0.014）。

4. MM+AL患者的器官受累情况：根据AL患者主要器官受累诊断标准及器官疗效标准[5]–[6]，42例MM+AL患者中累及肾脏8例（19.1％），5例缓解，3例进展；累及心脏6例（14.3％），2例缓解，4例进展；累及肝脏3例（7.2％），2例缓解，1例进展；累及周围神经1例，疗效评估为缓解；累及骨髓11例（26.2％）；累及皮肤29例（69.0％）；累及肺1例（2.4％）。根据梅奥2004分期，6例累及心脏患者中Ⅱ期3例，Ⅲa期2例，Ⅲb期1例。根据梅奥2012分期，6例累及心脏患者中1期1例，2期1例，4期4例。

5. 疗效评估：两组初诊MM患者在经过4个疗程的规范诱导治疗后行疗效评估，根据是否行自体造血干细胞移植分为移植组及未移植组，并进行相应巩固治疗。两组患者接受诱导治疗的方案、行自体造血干细胞移植比例的差异无统计学意义（表1）。患者疗效评估参照2017年修订版中国多发性骨髓瘤诊治指南[4]。MM组101例患者中29例疗效≥CR，36例VGPR，29例PR，4例SD，3例PD。MM+AL组38例患者中17例疗效≥CR，15例VGPR，4例PR，2例PD。MM+AL组患者ORR明显优于MM组患者（85.7％对79.7％，*P*＝0.049），MM+AL组达到VGPR以上疗效的患者比例也高于MM组患者（76.2％对55.1％，*P*＝0.019）。

6. 生存分析：截至 2020 年5月31日，4例失访。中位随访时间为24（0.25～41）个月，30个月时MM组累积生存率为83.04％，MM+AL组累积生存率为75.77％。中位PFS时间和OS时间均未达到（图1）。其中6例MM+AL组心脏受累患者的中位OS时间为13个月，与MM组和MM+AL组非心脏受累患者相比差异有统计学意义（*P*<0.001）。

**图1:**
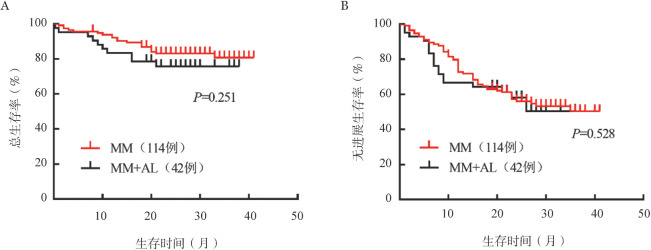
多发性骨髓瘤（MM）与MM合并轻链型淀粉样变性（MM+AL）患者的总生存（A）和无进展生存（B）曲线

两组患者根据是否进行自体造血干细胞移植分为四组：MM移植组、MM未移植组、MM+AL移植组、MM+AL未移植组，中位OS时间均未达到，仅MM未移植组达到中位PFS时间，为26个月。对于MM患者，移植组的PFS和OS均优于未移植组（*P*值均<0.05）（图2）。对于MM+AL患者，移植组的OS优于未移植组（*P*＝0.026）（图3）。

**图2:**
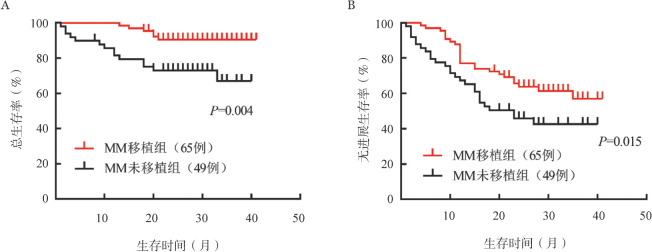
多发性骨髓瘤（MM）移植组与未移植组患者的总生存（A）和无进展生存（B）曲线

**图3:**
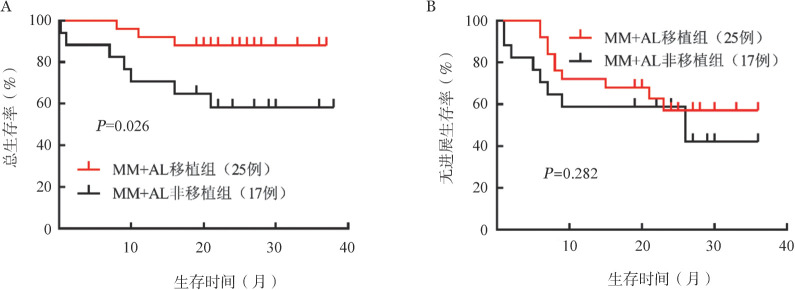
多发性骨髓瘤合并轻链型淀粉样变性（MM+AL）移植组与非移植组患者的总生存（A）和无进展生存（B）曲线

## 讨论

据报道，12％～15％的MM患者在初次诊断或病程后期会出现AL，但高达30％的患者在皮下脂肪、骨髓、心脏、肝脏和肾脏等其他重要器官的活检中出现亚临床轻链型淀粉样蛋白沉积[8]–[10]。也有文献提出，未被认识的AL可能与MM显著的死亡率和发病率有关，早期发现可能会改变MM患者的治疗[11]。

Abraham等[12]报道，AL与MM并存的患者预后较差，中位生存期为1.1年，而不伴AL的MM患者中位生存期为2.9年。另一项研究也指出，轻链型淀粉样蛋白沉积是MM的独立预后因素，患者的生存更差[13]。与此结论相反的是，Petruzziello等[14]的一项回顾性临床研究对166例MM患者平均随访近2年，并未发现刚果红染色阳性组和阴性组患者的生存有差异。Desikan等[15]在1997年的一项前瞻性临床试验中评估了84例既往从未接受过治疗或仅接受过最低限度治疗的MM患者，在治疗前进行皮下脂肪活检和骨髓刚果红染色，32例（38％）患者在≥1个部位发现了轻链型淀粉样蛋白，但PFS和OS的差异也无统计学意义。

本研究中MM合并AL的患者占26.3％，与已报道的研究结果一致[10],[12]–[13],[16]–[17]。MM+AL组的dFLC明显高于MM组（*P*＝0.033），与Usnarska-Zubkiewicz等[18]报道的结果一致。

在诱导治疗后的疗效方面，MM+AL组患者的ORR、≥VGPR率均优于MM组患者，即诱导治疗后，MM+AL组患者缓解率较MM组更高。但是两组患者PFS和OS的差异均无统计学意义，诱导治疗后进行自体造血干细胞移植能改善MM和MM合并AL患者的OS。再将MM+AL组分为累及心脏亚组和未累及心脏亚组，生存分析显示，MM+AL累及心脏组预后较差，中位OS时间仅13个月。因此在MM中常规进行淀粉样变筛查十分有必要，尤其是对于心脏受累者[19]。

本研究目前仍有不足之处，即纳入病例数相对较少，且随访时间太短。关于MM相关AL，目前仍需要扩大样本量、多中心临床数据及延长患者的随访时间，以进一步验证轻链型淀粉样蛋白的沉积对MM患者生物学、临床特征及预后的影响。
